# Salvaging Affymetrix probes after probe-level re-annotation

**DOI:** 10.1186/1756-0500-1-66

**Published:** 2008-08-19

**Authors:** Wim C de Leeuw, Han Rauwerda, Martijs J Jonker, Timo M Breit

**Affiliations:** 1 MicroArray Department & Integrative Bioinformatics Unit, Swammerdam Institute for Life Sciences, Faculty of Science, University of Amsterdam, Kruislaan 318, 1098 SM Amsterdam, the Netherlands

## Abstract

**Background:**

Affymetrix GeneChips can be re-annotated at the probe-level by breaking up the original probe-sets and recomposing new probe-sets based on up-to-date genomic knowledge, such as available in Entrez Gene. This results in custom Chip Description Files (CDF). Using these custom CDFs improves the quality of the data and thus the results of related gene expression studies. However, 44–71% of the probes on a GeneChip are lost in this re-annotation process. Although generally aimed at less known genes, losing these probes obviously means a substantial loss of expensive experiment data. Biologists are therefore very reluctant to adopt this approach.

**Findings:**

We aimed to re-introduce the non-affected Affymetrix probe-sets after these re-annotation procedures. For this, we developed an algorithm (CDF-Merger) and applied it to standard Affymetrix CDFs and custom Brainarray CDFs to obtain Hybrid CDFs. Thus, salvaging lost Affymetrix probes with our CDF-Merger restored probe content up to 94%. Because the salvaged probes (up to 54% of the probe content on the arrays) represent less-reliable probe-sets, we made the origin of all probe-set definitions traceable, so biologists can choose at any time in their analyses, which subset of probe-sets they want to use.

**Conclusion:**

The availability of up-to-date Hybrid CDFs plus R environment allows for easy implementation of our approach.

## Findings

### Background

Affymetrix GeneChips are widely used for transcriptome analysis. This microarray platform is based on the concept of a set of 11–22 probes representing each gene. However, the genomic knowledge used by Affymetrix for the original probe-set definitions becomes increasingly outdated. This compels the redefinition of these probe-sets at probe level using current genomic knowledge. Several studies addressed this issue [1-4]. In these approaches, the original probe-set definitions are discarded and all probes are recomposed into new probe-sets by mapping each probe via their sequence to unique genes available in one or more well-defined genomics resources (Entrez Gene, Refseq, Ensembl). The approaches differ with respect to the used genomic resources and genetic elements (genes or transcripts), as well as the chosen parameters, such as minimum number of probes per probe-set, percentage sequence similarity, and so on. In order to make these new probe-sets available to life scientists, new Affymetrix compatible custom Chip Description Files (CDFs) are produced that contain probe-sets, each consisting of a few to several hundreds probes.

Because these custom CDFs are based on the latest genomic knowledge, the newly defined probe-sets perform better in gene-profiling studies [5,6] or allow for more reliable cross-platform comparison of gene expression [4]. Also, since genes are uniquely represented in a custom CDF, bias towards genes represented by multiple probe-sets is avoided in gene-set enrichment. With these advantages, one would expect these probe re-annotation approaches to be readily embraced by the research community. Although there is an increasing interest for this issue, still a limited number of studies actually used such an approach [7-11]. One of the reasons might be that custom CDFs require a somewhat more advanced bioinformatics environments, both in software and expertise.

As we experience in practice, biologists do not accept the low percentages of probes left after re-annotation (Table 1). Given the costly nature of Affymetrix based experiments, they will not easily discard 44–71% of their data, even though they know that the quality of the annotation of these probe sets is poor. They want to keep these probes sets because commonly their studies are not focussed only on well-established genes, but also on the involvement of new (poorly-annotated) genes. Quite often even their favourite genes are absent in the custom CDFs. In fact, some biologists fix the loss of their favourite genes by reintroducing them using the old Affymetrix probe-set definition [10]. This entails two separate, but redundant analyses that are difficult to compare. This motivated us to merge these two analyses from the start, by salvaging as many probe-sets as possible that are lost during probe re-annotation. To illustrate our point, in the study described in [10], a number of genes involved in epileptogenic development, such as Kncd2, a Potassium channel protein and CD11b/c (OX42), a marker for microglial activation are absent in the Brainarray CDF. These genes were added to the analyses and proved to be important in the biological study at hand. Currently, analyses of several Affymetrix experiments are in progress using our Hybrid CDFs.

**Table 1:** Percentage of probe usage after re-annotation

**Organism**	**GeneChip**	**Affymetrix**	**Brainarray **[1]	AffyProbe-Miner * [3]	**Hybrid**
H. sapiens**	HG-U133_Plus_2	604,258	40%	56%	94%
M. musculus	Mouse430_2	496,468	49%	56%	94%
R. norvegicus	Rat230_2	342,410	40%	29%	94%
B. taurus	Bovine	265,627	44%	29%	93%
D. rerio	Zebrafish	249,752	39%	46%	88%

* Gene Consistent RefSeq plus GenBank, min. probe-set size = 5.

** GeneAnnot [2]: 58% probe usage.

Here we present a procedure, CDF-Merger, to formally compose such hybrid probe-set definitions based on the Brainarray approach by [1]. Thus, we generate Hybrid CDFs in which Entrez gene defined genes are uniquely represented and probe usage is maximized by reusing the information provided by Affymetrix, as long as there is no conflict with the Brainarray probe-sets. Given the difference in annotation quality between custom CDF annotation and original Affymetrix annotation, we made the origin of probe-set definitions in the Hybrid CDFs traceable by extensions on probe-set ids. In this way, biologist can choose at any time during their analysis which gene set they would like to use. Hybrid CDFs are Entrez based, i.e. gene-centric. If at some point in the analysis, a more transcript-centric approach is needed, tools such as ADAPT [12] can be used to link identifiers in the Hybrid CDF by their Affymetrix or Entrez Gene IDs via Ensembl to their transcripts.

### Description

Hybrid probe-set definitions are generated using Brainarray CDFs [13], Affymetrix NetAffx Annotation Files[14], and NCBI Entrez Gene Info Files[15]. The original procedure to generate custom Brainarray Entrez probe sets entails the mapping of each -perfect match- Affymetrix probe to the appropriate Entrez Gene annotated target sequences. Probes with more than one or no perfect hits are removed and each final probe set must contain at least three probes [1].

The following CDF-Merger algorithm aims to salvage a maximum number of probes from Affymetrix GeneChips that are lost by Brainarray re-annotation:

a. Rename the Brainarray defined probe-sets from a specific Brainarray CDF to allow future traceability: ***Hybrid probe-set id: atd_ [Entrez id]***

b. Check each Brainarray defined probe-set for probes also present in other Brainarray probe-sets. *If so, mark such a probe-set with extension _d*.

c. Keep the Affymetrix defined probe-set names from the relevant Affymetrix NetAffx Annotation File to allow future traceability: Hybrid probe-set id: AFFX-* or *_at*(= original Affymetrix id)*

d. Check each Affymetrix defined probe-set and discard those with more than two probes that are also used in the Brainarray probe-sets.

e. Check if in the remaining probe-sets from step d, one or two probes are also used in Brainarray probe-sets. If so, keep the probe-set but remove these probes. *If so, mark the Hybrid probe-set _1 or _2, respectively*.

f. Use the NCBI Entrez Gene Info File to check if in the Affymetrix NetAffx Annotation file, the remaining Affymetrix probe-sets have exactly one valid Entrez id. If not, remove all Entrez ids from the annotation of these Affymetrix probe-sets.

g. Check whether the Entrez id of the remaining Affymetrix probe-sets with one valid Entrez id also occurs in the Brainarray CDF. If so, remove this Entrez id from the annotation of these Affymetrix probe-sets.

h. Rename all Affymetrix probe-sets retained in steps f and g, to allow future traceability: ***Hybrid probe-set id: atm_ [Entrez id]***

i. Merge the Affymetrix probe-sets from step h with the same Entrez id. *Mark merged probe-sets with extension _m*.

j. Compile a Hybrid CDF and R environment with these hybrid probe-set definitions and associated annotations.

**Figure 1:**
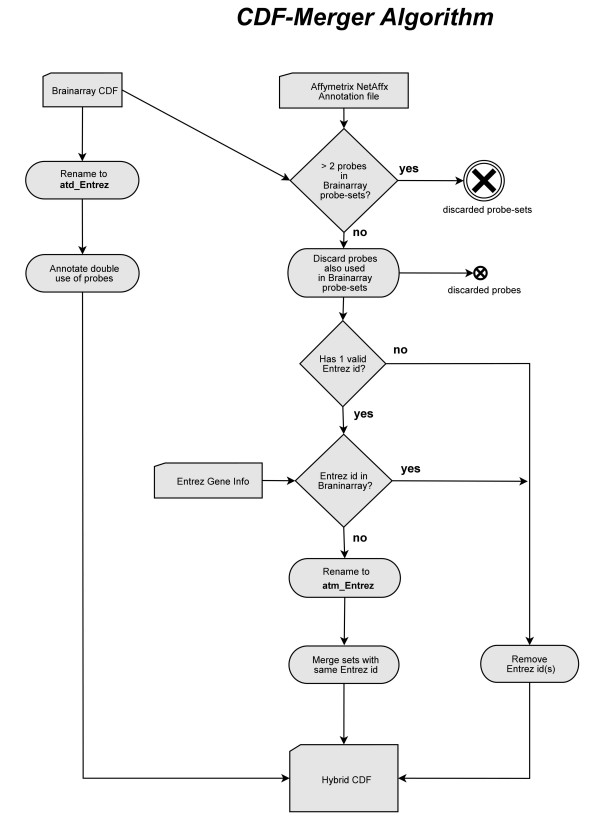
**CDF-Merger Algorithm**. Schema of the CDF-Merger algorithm.

A flowchart of this CDF-Merger algorithm can be found in supplementary information (Figure 1). The Hybrid CDFs, the source code of the CDF-Merger algorithm, and instructions on use of these environments in R are online available.

### Concluding Remarks

The motivation for the development of our CDF-Merger algorithm and the resulting Hybrid CDFs, came from complaints of biologists that disliked the high percentages of Affymetrix GeneChip probes lost during re-annotation. To illustrate their point, we compared probe usage of two recently published re-annotation algorithms for five commonly used Affymetrix GeneChips (Table 1). In the listed alternative probe-set definitions, the amount of data at the probe level is dramatically decreased compared to the original Affymetrix probe-set definitions, because 44% to 71% of probes are discarded. In contrast, because we salvage the far majority of lost probes with our CDF-Merger approach, ultimately only 6% to 12% of all probes are discarded. These mostly represent probes from Affymetrix defined probe-sets for genes that are already covered by Brainarray defined probe sets. The source code of the CDF-Merger algorithm, definition origin, and probe-set sizes in all Hybrid CDFs [see Additional File 1 Table S1], a table of probe distribution [see Additional File 2 Table S2] are online available and allow easy updating of the Hybrid CDFs. Although we based our CDF-Merger algorithm on the Brainarray re-annotation, our approach is, with minor adjustments, also applicable to the AffyProbeMiner and GeneAnnot reannotations.

It is obvious that re-introducing Affymetrix probe-sets will dilute the data quality enhancement from the original custom re-annotation procedures, which are based on up-to-date genomic knowledge. However, our hybrid approach, which is in essence a practical compromise, aims to get the concept of re-annotated probe-sets more widely accepted in the biology community. For this, we made the whole CDF-Merger algorithm traceable, so it is clear where each probe-set originates from. As such, biologists and bioinformaticians can decide at any time during their extensive bioinformatics analyses whether they would like to use a) limited, but well-established data, or b) all data, which contains more ambiguity, but also more unknown genes and transcripts. The impact of using the Hybrid CDFs strongly depends on the character of a microarray study and the applied bioinformatics analysis methods. Altogether, we think that our hybrid CDF-Merger approach, which salvages most lost probes after probe-level re-annotation, combines the best of both worlds to enable the often discovery-driven omics experimentation.

## Availability and requirements

• **Project name**: Hybrid CDF

• **Project home page: **

**Programming language: **R

• **Other requirements: **BioConductor affy library

• **Source code:**

• 

• **CDF files, R cdf environments, R annotation environments: **

All files will be updated after Brainarray release.

## Competing interests

The authors declare that they have no competing interests.

## Authors' contributions

WdL specified and implemented the CDF-Merger algorithm.

HR, MJ and TB all worked on the specification of the CDF-Merger algorithm and adapted it by discussing applicability of it with biologists.

The authors wish it to be known that, in their opinion, WdL and HR should be regarded as joint First Authors.

## Supplementary Material

Additional file 1 Definition origin and probe-set sizes Brainarray version 10. Click here for file

Additional file 2 Hybrid probe-set distribution using Brainarray version 10. Click here for file
